# Cannabidiol Combination Enhances Photodynamic Therapy Effects on MCF-7 Breast Cancer Cells

**DOI:** 10.3390/cells13020187

**Published:** 2024-01-18

**Authors:** Dimakatso Mokoena, Blassan P. George, Heidi Abrahamse

**Affiliations:** Laser Research Centre, Faculty of Health Sciences, University of Johannesburg, P.O. Box 17011, Doornfontein 2028, South Africa; 200905898@student.uj.ac.za (D.M.); blassang@uj.ac.za (B.P.G.)

**Keywords:** cannabidiol, MCF-7 cells, breast cancer therapy, PDT, nanotechnology, hypericin, cell death

## Abstract

*Cannabis sativa* is a well-known plant for its psychoactive effects; however, its many derivatives, such as Cannabidiol (CBD), contain several therapeutic applications. Tetrahydrocannabinol (THC) is the main cannabis derivative responsible for psychoactive properties, while CBD is non-psychotropic. For this reason, CBD has been more exploited in the last decade. CBD has been connected to multiple anticancer properties, and when combined with photodynamic therapy (PDT), it is possible to eradicate tumors more effectively. In this study, CBD was utilized to treat MCF-7 breast cancer cells, followed by in vitro PDT combination therapy. Conventional breast cancer treatment modalities such as chemotherapy, radiotherapy, etc. have been reported for inducing a number of undesirable side effects, recurrence of the disease, and low quality of life. In this study, cells were exposed to varying concentrations of CBD (i.e., 1.25, 2.5, 5, 10, and 20 μg/mL) and incubated 12 and 24 h after treatment. The optimal doses were then used in combination therapy. Morphology and biochemical assays, including lactate dehydrogenase (LDH) for membrane integrity, adenosine triphosphate (ATP) for viability, and trypan blue exclusion assay for viability, were used to examine cellular responses after treatments. The optimal concentration was then utilized in Hypericin-Gold nanoparticles mediated PDT combination. The results revealed that, in a dose-dependent manner, conventional morphological characteristics of cell death, such as vacuolization, blebbing, and floating were observed in treated cells. The biochemical responses demonstrated an increase in LDH, a decrease in ATP, and a reduction in viability. This study demonstrated that CBD induces cell death in MCF-7 breast cancer cells cultured in vitro. The immunofluorescence results of combination therapy indicated that cell death occurred via apoptosis. In conclusion, this study proposes that the CBD and PDT combination therapy is effective in killing MCF-7 breast cancer cells in vitro by induction of apoptosis.

## 1. Introduction

Cannabis is the most widely used illicit substance worldwide, and its use is associated with psychoactive properties. However, cannabis also possesses a number of beneficial properties that should be investigated. Anticancer and anti-inflammatory properties are among them. Over 400 chemical units make up the cannabis plant, of which more than 60 are cannabinoids [1]. In ancient India, it was used in ayurvedic medicine to relieve pain, anxiety, and nausea, to relax muscles, to enhance sleep and appetite, and to induce euphoria [2]. The first discovered exogenous substances, also known as phytocannabinoids, are cannabidiol (CBD) and tetrahydrocannabinol (THC). Due to its psychoactive effects, THC has always received more attention than CBD [2,3]. Until the discovery of the endocannabinoid system, however, other cannabinoids, including CBD, were not recognized. Cannabis is primarily recognized for its psychoactive effects. It has several beneficial properties that have not been fully investigated nor comprehended for decades, despite the fact that only its negative aspects have been studied. It has always been reduced to illicit recreational use and pharmaceutical exploitation to produce drugs. 

Cannabinoids, the various derivatives of the cannabis plant, contribute to the plant’s diverse characteristics. Each derivative has unique properties, such as antitumor, anti-inflammatory, psychoactive, appetite stimulation, sleep improvement, etc. It is necessary to investigate cannabis and its derivatives further. *Cannabis sativa*, *Cannabis ruderalis*, and *Cannabis indica* are the three most widely recognized species of Cannabis plant. *Cannabis sativa* (*C. sativa*) is a widely cultivated and abundant species that contains more than 60 cannabinoids. Tetrahydrocannabinol (THC) is a popular cannabinoid due to its psychoactive effects [4,5]. Cannabinoids’ antitumor properties include the activation of cell death, inhibition of tumor metastasis and angiogenesis, and inhibition of tumor cell proliferation, according to the reports [6,7]. Cannabis and medicinal cannabinoids showed many potential benefits; patients with chronic pain treated with cannabis or cannabinoids are more likely to experience a clinically significant reduction in pain symptoms. Short-term use of oral cannabinoids improves patient-reported multiple sclerosis (MS)-related spasticity symptoms. Cannabinoids are effective antiemetics, help to control chemotherapy-induced nausea and vomiting [8].

CBD inclusion is a promising integrative strategy to cancer care. CBD appears to have therapeutic potential in the treatment of cancer, according to research. CBD appears to interfere with pathways involved in cancer pathogenesis, according to preclinical research. Preclinical and clinical studies demonstrate some benefit, alone or in combination with the other important phytocannabinoids, in treating cancer-related pain, anxiety and depression, sleep disorders, nausea and vomiting, and oral mucositis. CBD may improve conventional treatments such as chemotherapy and radiation, as well as protect against neurological and organ damage [9]. By binding to endocannabinoid receptors, cannabinoids induce the activity of enzymes that influence various physiological and pathological processes [7]. The phytocannabinoids, such as THC, CBD, and cannabinol (CBN), naturally generated endocannabinoids, and synthetic cannabinoids are the common types of cannabinoid receptors [10,11]. The binding of cannabinoids to specific cell surface receptors has also been observed to increase tumor cell oxidative stress by elevating ROS levels above the cancer cells’ threshold, resulting in a disturbed cancer cell redox balance [12,13,14]. It is well known that cancer cells utilize ROS signaling for survival, migration, and proliferation by upregulating nuclear factor erythroid 2 related factor 2 (NRF2), which results in glutathione peroxidase and peroxiredoxin enzyme activation for glutathione (GSH) synthesis and antioxidant activation [15]. 

Photodynamic therapy (PDT) is a clinically approved, minimally invasive treatment for the treatment of cancer by exerting selective toxicity on the cancer cells. PDT involves the administration of phototoxic agents called Photosensitizers (PS) with specific wavelength light in the presence of molecular oxygen. Recent research has shown that PDT has advantages and potential to become integrated into the mainstream cancer treatment. Using natural compounds such as phytochemicals has shown potential in improving PDT outcomes with reduced side effects [16,17]. Hypericin PS has been used in PDT application in various cancer cells in vitro, including skin and breast cancers. To investigate the improved effective delivery of PS, in this study, Hypericin PS was conjugated with gold nanoparticles. Breast cancer is commonly referred to as a category of diseases due to the occurrence of many biological subtypes with diverse molecular profiles and clinicopathological features. Different factors for breast cancer risk development or progression, such as gender, age, and other hereditary factors, have been linked in epidemiological research. Because of its high incidence and fatality rates, breast cancer is a major global health concern among women. Even with adjuvant chemotherapy, the five-year survival rate for metastatic breast cancer is less than 30%. Breast cancer is more common in high-income countries than in low-income countries, indicating a link with globalization. The MCF-7 cell line was the first hormone-responsive human breast cancer cell line that was widely employed for tumor biology and study of mechanism of action [18]. Even though there are many breast cancer cell lines based on the breast cancer type available, we adopted the commonly used hormone-responsive breast cancer cell line MCF-7 in this study, as it is well-characterized and frequently used in cancer research. This study was designed to investigate the effect of CBD and PDT combination therapy to treat MCF-7 breast cancer cells in vitro.

## 2. Materials and Methods

### 2.1. MCF-7 Cell Culture and CBD Treatment

MCF-7 (ATCC^®^ HTB-22™) cell lines from American Type Culture Collection (ATCC) were cultured at a concentration of 3 × 10^5^ in culture plates with a diameter of 3.4 cm. Cells were grown in 3 mL of prewarmed, complete medium containing Dulbecco’s Modified Eagle’s Medium (DMEM), 5% Fetal Bovine Serum (FBS), 1% Pen-Strep, and 1% Amphotericin B. This was followed by 37 °C incubation in 5% CO_2_ and 85% relative humidity. After 4 h of attachment, cells were washed with pre-warmed Hank’s Balanced Salt Solution (HBSS) and treated with CBD (Sigma-Aldrich, 90899-1 mL, St. Louis, MO, USA) at the following concentrations: 1.25, 2.5, 5, 10, and 20 µg/mL from a 5 mg/mL stock solution in 3 mL of complete medium. Cells were then incubated for 12 and 24 h before checking cellular responses. Cells that were not treated with CBD served as controls.

### 2.2. CBD Dose Response

Cellular morphology was observed 12 and 24 h after CBD treatment using an inverted light microscope (Wirsam Scientific, Olympus CKX41, Johannesburg, South Africa). Images were acquired using the built-in camera and visualized using the CellSens imaging software 2.3 version. The Lactate Dehydrogenase (LDH) cytotoxicity assay was conducted using the CytoTox 96^®^ Non-Radioactive Cytotoxicity Assay (Anatech: Promega, Madison, WI, USA, PRG1780). The assay detected the amount of LDH in the culture media after the treatment of cells using spectrophotometric analysis of LDH at 490 nm (Perkin-Elmer, Victor Nivo^TM^, Johannesburg, South Africa). This was accomplished by adding equal volumes of culture media and substrate reagent mix to a 96-well plate, followed by 30 min of room temperature incubation in the dark. All values obtained were calculated against the maximum cytotoxicity obtained by seeding the same number of cells used for experimental groups in a 3.4 cm diameter plate and lysing them 45 min before the assay was performed.

On cultured cells, the Adenosine Triphosphate cell viability assay was conducted using the CellTiter-Glo Luminescence Cell Viability Assay (Anatech, Promega, G7570). The assay measured the luminescence signal produced by the conversion of ATP to adenosine monophosphate (AMP) through the activity of the enzyme luciferase to determine the amount of ATP in treated cells. On the Victor-3 (Perkin-Elmer, Victor Nivo^TM^, Johannesburg, South Africa) multi-plate reader, the luminescence results were displayed in relative light units (RLU). The trypan blue assay was performed to distinguish viable cells from non-viable cells based on their inability to absorb the trypan blue pigment. After treatment, cells were removed from a cell culture dish and added in equal volumes to 0.4% trypan blue dye. This was followed by a mild mix and a 2 min incubation at room temperature. The slide was then inserted into the countess automated benchtop cell counter (Invitrogen Countess^®^ II FL automated cell counter) to be read. 

### 2.3. Combination Therapy

The optimal CBD concentration was used in combination with PDT augmented by gold nanoparticles. In this combination therapy, the optimal concentrations determined in a previous study were used [16]. There were three treatment groups: pre-PDT (CBD first), post-PDT (PDT first), and simultaneous treatment (combination therapy). For pre-PDT, cells were treated with CBD after 4 h attachment and incubated for 12 h, after which the optimum established concentration of nanoconjugate (Hypericin-AuNP) was added and incubated for 12 h. Similarly, for post-PDT, cells were treated with PDT first, then CBD; the treatment follows the same procedure as the pre-PDT except that PDT was performed first, then followed by CBD treatment. For simultaneous treatment, cells were treated with both CBD and the Hypericin-AuNP nanoconjugate simultaneously under similar treatment conditions to those of pre- and post-PDT. We used the diode laser with continuous wave emission wavelength of 594 nm and energy density 5 J/cm^2^ (irradiation time 8 min 18 s) for irradiation. In a CO_2_ incubator, cells were inoculated in 3.4 cm^2^ culture plates and allowed to attach for 4 h. Morphology, ATP, and LDH biochemical assays were used to observe the effects of the combination therapy, followed by immunofluorescence 12 h after treatment. 

#### Immunofluorescence

To qualitatively evaluate the cell death induced by the combination therapy, the apoptotic pathway markers Cytochrome c, Bcl-2, Bax, p53, and PARP were investigated. 3 × 10^5^ cells were inoculated on coverslips in culture plates with a diameter of 3.4 centimeters. After treatment, cells were stained with specific primary antibodies and fluorochrome-conjugated secondary antibodies, beginning with rinsing cells twice with 1× phosphate-buffered saline (PBS), followed by fixing cells for 15 min at room temperature with 1 mL 4% paraformaldehyde in 1× PBS. The cells were then washed three times with PBS-T (0.1% Tween-20 in 1× PBS) wash buffer, permeabilized for 15 min at room temperature with 0.5% Triton X-100 in 1× PBS (permeabilization solution) and rinsed three times. The cells were then blocked for 1 h at room temperature with 1 mL of 1% BSA (1% Bovine Serum Albumin in 1× PBS) blocking solution to prevent nonspecific antibody binding. Anti-Bax (QI213591, Life Technology, Carlsbad, CA, USA), Anti-Bcl-2 (QF215245, Life Technology), Anti-p53 (SC-99, Santa Cruz Biotechnology, Dallas, TX, USA), 10 g/mL Anti-Cytochrome c (MAB1420, Santa Cruz Biotechnology), and Anti-PARP-1 (MAB1420, Santa Cruz Biotechnology) were used to stain the cells for 1 h at 37 °C. The cells were then washed three times and stained for 1 h at room temperature in the dark with the secondary antibodies at 1:200 concentrations in PBS-T goat anti-mouse FITC conjugated (D2706, Santa Cruz Biotechnology) and donkey anti-mouse NL557 conjugated (NL002, R&D Systems, Minneapolis, MN, USA, Whitehead Scientific, Cape Town, South Africa). The cells were flushed three times with wash buffer and counterstained for 5 min with 1 µg/mL DAPI (4’,6-diamidino-2-phenylindole). The coverslip was removed from the 3.4 cm plate and inverted onto a glass slide with a drop of fluoromount aqueous mounting medium after the cells were washed three times. The samples were observed using the Carl Zeiss Axio Observer Z1 live imaging fluorescent microscope and ZEN 3.1 (ZEN pro) software.

### 2.4. Statistical Analysis 

The experiments were conducted three times (*n* = 3) and statistical analysis was performed using SigmaPlot software version 14.0. A Student’s *t*-test was performed to analyze the statistical significance between the control (untreated cells) and experimental groups. Significance is reported as *p* < 0.05 (*), *p* < 0.01 (**), and *p* < 0.001 (***). Standard error is indicated by error bars on bar graphs.

## 3. Results

### 3.1. Morphology

**Figure 1:**
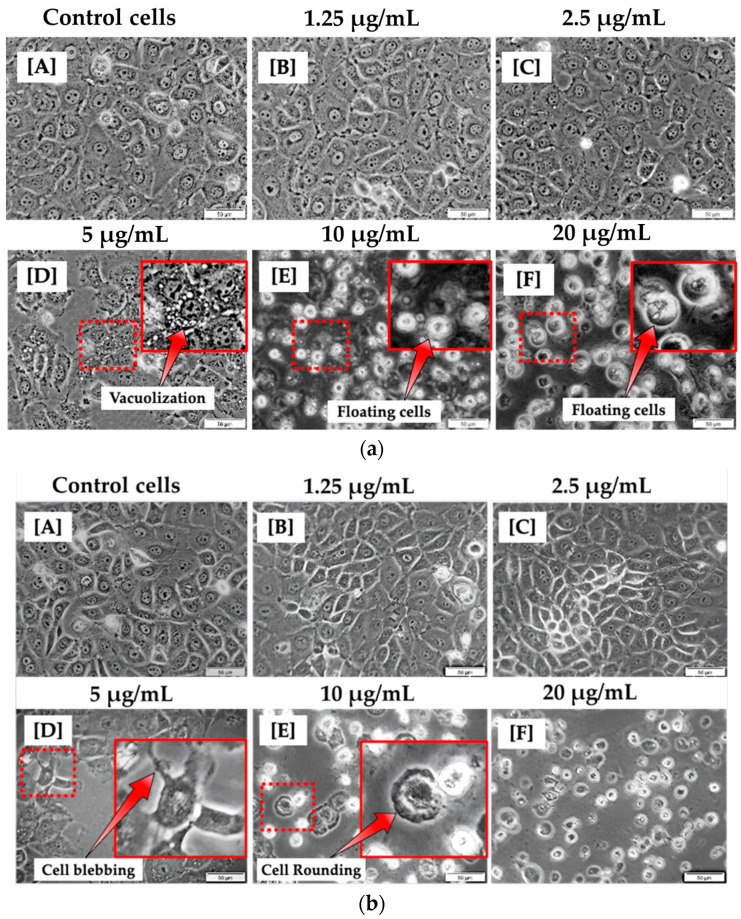
Morphological analysis of MCF-7 cells 12 h (**a**) and 24 h (**b**) post treatment with varying concentrations of CBD (A–F). No morphological changes were observed in MCF-7 cells treated with 1.25 and 2.5 μg/mL (B,C). However, morphological changes were observed in MCF-7 cells treated with 5, 10, and 20 μg/mL of CBD (D–F) when compared to control cells (A). (100× magnification and scale bar: 50 μm).

At 12 h of incubation, the morphological appearance of cells treated with lower concentrations of CBD, such as 1.25 and 2.5 μg/mL, was comparable to that of untreated cells. This suggests that CBD at modest concentrations may have no effect on MCF-7 cells. At concentrations of 5, 10, and 20 μg/mL, significant differences were observed in comparison to untreated control cells. As shown in Figure 1a, this is evidenced by the presence of vacuoles in the cytoplasm and floating cells. At 24 h of incubation, the morphology of cells treated with 1.25 and 2.5 µg/mL CBD did not differ significantly from untreated cells. As shown in Figure 1b, at 5, 10, and 20 µg/mL CBD concentrations, cells exhibited evident morphological differences, including blebbing, vacuolization, cell rounding, and floating, compared to control cells.

### 3.2. LDH Membrane Integrity

**Figure 2:**
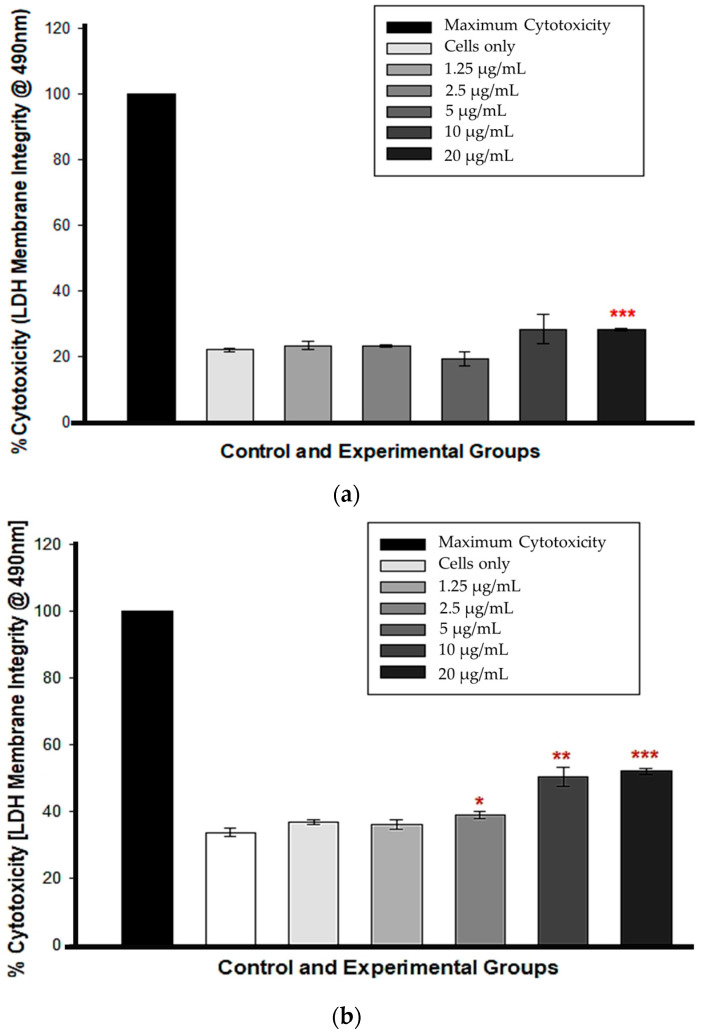
LDH membrane integrity test following a 12 h (**a**) and 24 h (**b**) CBD incubation. * *p* < 0.05, ** *p* < 0.01 and *** *p* < 0.001 (SEM) denote statistical significance.

At 12 h, cytotoxicity was observed only in cells treated with 20 µg/mL CBD (*** *p* < 0.001), whereas cells treated with 1.25, 2.5, 5, and 10 µg/mL CBD exhibited no statistically significant LDH presence in comparison to untreated control cells, indicating no cytotoxicity (Figure 2a). At 24 h, the percentage increase in LDH cytotoxicity was dose-dependent and statistically significant from 5 to 20 µg/mL CBD concentrations, *p* < 0.05, 0.01 and 0.001, respectively. Figure 2b shows that at concentrations of 1.25 and 2.5 µg/mL, there was no statistical significance compared to the control cells. 

### 3.3. ATP Luminescence

**Figure 3:**
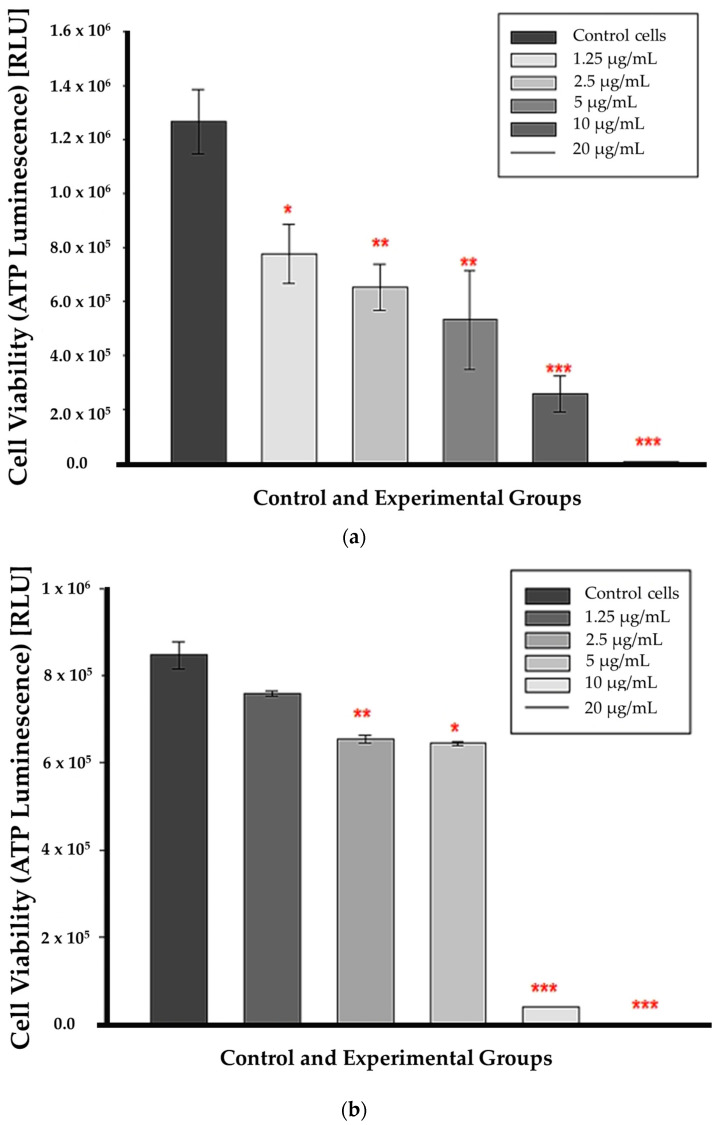
ATP luminescence assay results after a 12 h (**a**) 24 h (**b**) incubation with CBD. Significance is denoted as * *p* < 0.05, ** *p* < 0.01 and *** *p* < 0.001 (SEM).

At 12 h of incubation, ATP levels were substantially lower in all CBD-treated cells than in untreated control cells. This suggests that CBD affected cell viability from the lowest to the highest concentrations as early as 12 h post-treatment, as shown in Figure 3a. ATP was markedly decreased in cells treated with CBD at concentrations of 2.5, 5, 10, and 20 µg/mL after 24 h of incubation (*p* < 0.01, *p* < 0.05, *p* < 0.001, respectively); (Figure 3b). At 1.25 µg/mL, ATP levels did not decrease significantly compared to untreated control cells. The *p* value was 0.112, indicating that the differences in mean values between the treated group and the control cells were insufficient to rule out the possibility that the observed differences were due to random sampling error or variation.

### 3.4. Trypan Blue Assay

**Figure 4:**
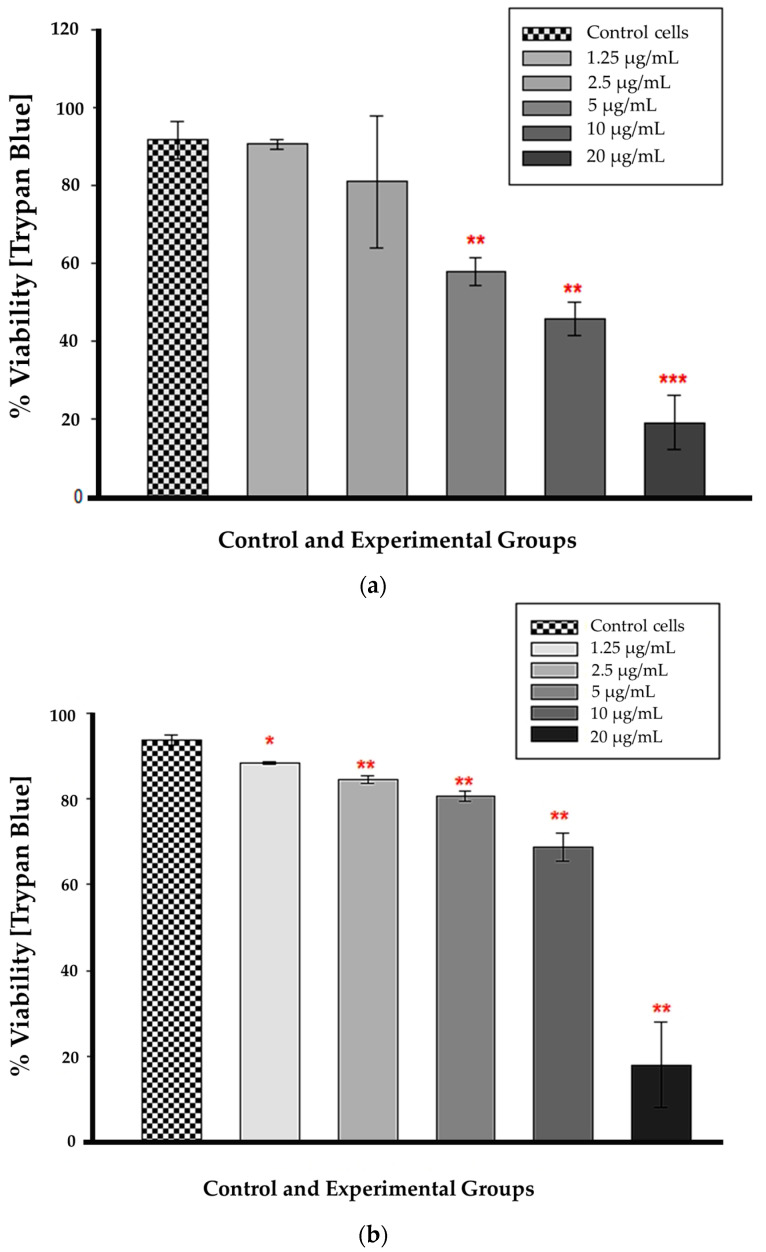
Trypan blue viability assay 12 h (**a**) and 24 h (**b**) after CBD treatment. * *p* < 0.05, ** *p* < 0.01 and *** *p* < 0.001 (SEM) denote statistical significance.

Statistical significance (*p* < 0.01 and *p* < 0.001) was observed at 12 h for CBD concentrations ranging from 5 to 20 µg/mL in comparison to untreated control cells (Figure 4a). At these concentrations, cellular viability was substantially reduced, and the difference from control cells was greater than would be expected by chance. The concentrations of 1.25 and 2.5 µg/mL had no statistical significance when compared to untreated control cells (*p* = 0.851 and 0.579, respectively). The large error bar displayed in the 2.5 µg/mL bar indicates that the data values had a greater variation than the mean. Figure 4b demonstrates that at 24 h, all CBD-treated cells were statistically significant compared to untreated control cells. At 1.25 µg/mL, *p* was less than 0.05, and from 2.5 to 20 µg/mL, *p* was less than 0.01, indicating that the mean difference between treated and untreated cells was greater than expected by chance. At 24 h, the CBD-treated cells had a lower percentage of viable cells compared to the untreated control cells.

### 3.5. Combination Therapy

#### 3.5.1. Morphology

**Figure 5:**
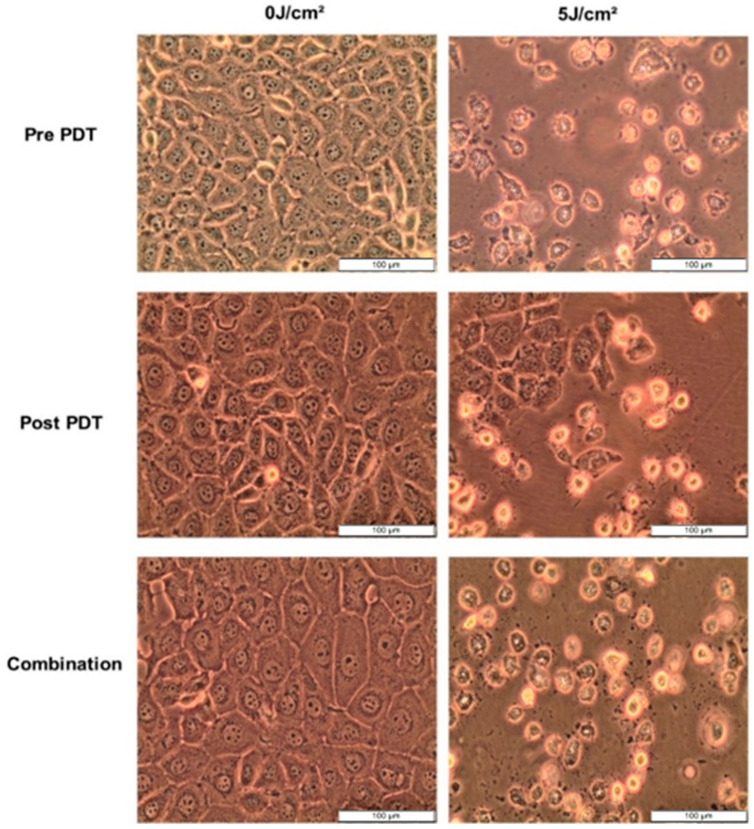
Cellular morphology of MCF-7 cells observed at 100× magnification prior to PDT, 12 h after CBD, Hypericin-AuNP PDT and in conjunction with Combination Therapy.

After 12 h treatment with 5 µg/mL CBD and 7.6 µM Hypericin-AuNP mediated PDT at 5 J/cm^2^, morphology changes were observed. Pre-PDT refers to cells treated with CBD alone, post-PDT refers to cells treated with PDT first followed by CBD treatment, and combination refers to cells treated with CBD and Hypericin-AuNP mediated PDT simultaneously. Compared to untreated control cells, treated cells exhibited visible morphological changes, as shown in Figure 5; these alterations are characterized by shrinking, rounding, floating, and cellular debris in the background. These characteristics are indicative of cell demise caused by the treatments.

#### 3.5.2. LDH

**Figure 6:**
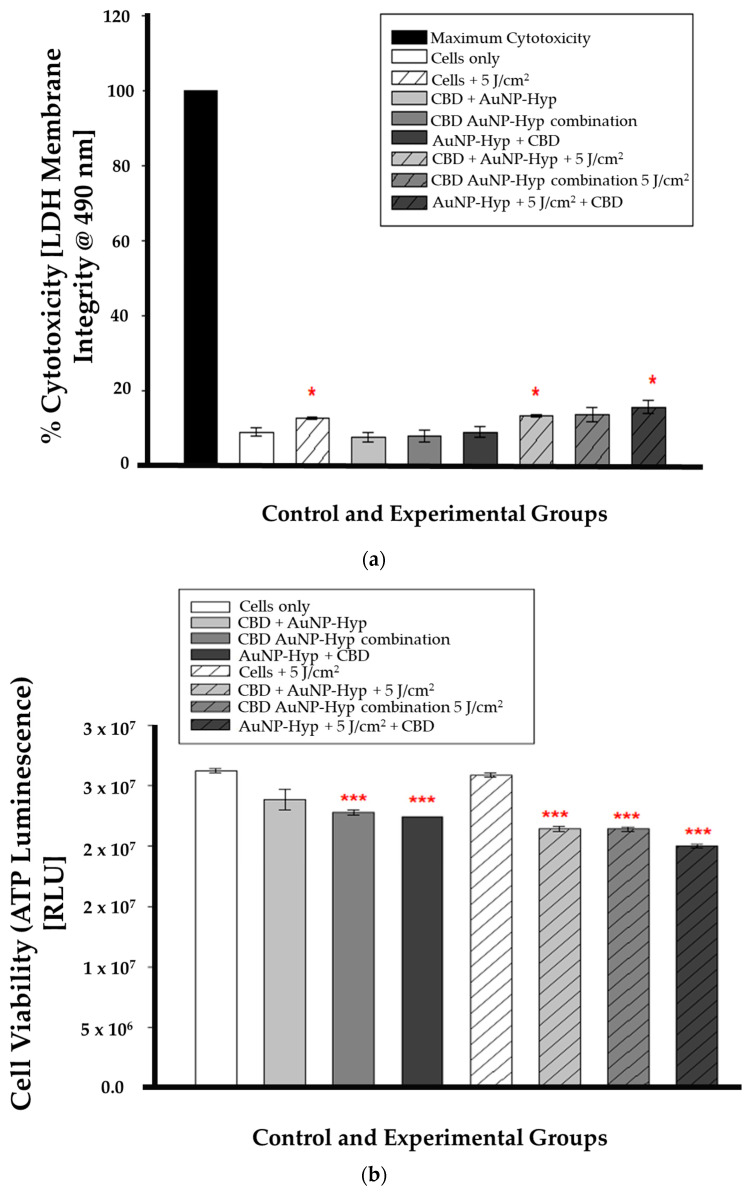
(**a**) LDH cytotoxicity after 12 h incubation following CBD and PDT with a 5 J/cm^2^ laser fluence. Significance is denoted as * *p* < 0.05 (SEM). (**b**) ATP luminescence after 12 h incubation following CBD and PDT with a 5 J/cm^2^ laser fluence. Significance is denoted as *** *p* < 0.001 (SEM).

In combination therapy, the LDH assay results were statistically significant (* *p* < 0.05) in irradiated cells (cells + 5 J/cm^2^), pre-PDT CBD-treated cells (CBD + Hypericin-AuNP 5 J/cm^2^), and post-PDT CBD-treated cells (Hypericin-AuNP 5 J/cm^2^ + CBD) compared to control cells, as shown in Figure 6a. While Figure 6b is a demonstration of the ATP luminescence assay results which were statistically significant (*** *p* < 0.001) in all experimental groups except for the cells treated with 5 J/cm^2^ of radiation (5 J/cm^2^ + cells).

#### 3.5.3. Immunofluorescence

**Figure 7:**
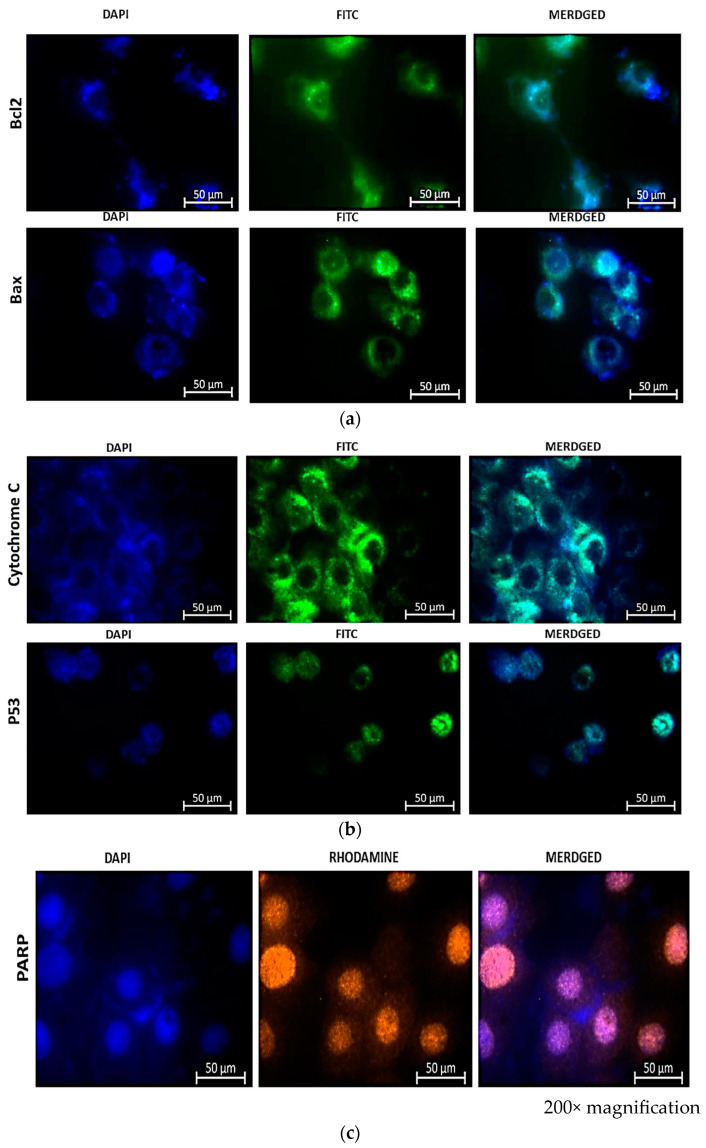
Immunofluorescence microscopy of MCF-7 cells reveals the presence of apoptotic proteins Bax and Bcl-2 (**a**), Cytochrome c and p53 (**b**), and PARP-1 (**c**) in combination therapy with CBD and Hypericin-AuNP. Twelve hours after combination therapy, cells were stained with FITC (green) and nuclei were counterstained with DAPI (blue).

Immunostaining of treated cells for Bax and Bcl-2 (FITC), cytochrome c and p53 (FITC), and PARP (orange), as shown in Figure 7, revealed the expression of apoptotic proteins following combination therapy treatment. The presence of fluorescence in combined images indicated the presence of proteins and, consequently, the activation of the apoptotic pathway induced by the combination therapy.

The immunofluorescence (IF) results shown in Figure 7a reveal the presence of Bcl-2 and Bax stained with FITC (green) and DAPI (blue) in the nuclei of the cells. Bcl-2 has a weaker signal than Bax, indicating that the cells are undergoing apoptosis. An enhanced Bcl-2 signal would indicate that the activity of Bcl-2 is inhibiting apoptosis, given that Bcl-2 is known to prevent programmed cell demise. It accomplishes this by increasing the membrane potential, thereby promoting cell survival. This also implies that an increased Bcl-2 signal would prevent the release of cytochrome c, thereby inhibiting apoptosis. The immunofluorescence signal of Bax is greater than that of Bcl-2, indicating that the cells are undergoing cell growth arrest, which results in the release of cytochrome c and the expression of p53 as shown in Figure 7b. The indirect rhodamine (Orange) staining of treated MCF-7 cells for PARP-1 (poly ADP-ribose polymerase) indicated the expression of the protein. PARP-1 is a PARP fragment that is only detected in the presence of DNA fragmentation and, consequently, cell demise. In the absence of PARP-1, cells are, therefore, undergoing irreversible cell demise. This is indicated by the orange color in Figure 7c.

## 4. Discussion

This study investigated the direct anticancer effects of CBD, a chemotherapeutic agent, on MCF-7 breast cancer cells. The morphology results of cells treated with CBD at concentrations ranging from 5 to 20 μg/mL for 12 h revealed an altered appearance. 24 h after incubation, the morphology of MCF-7 cells remained altered, with indications of cell death including cytoplasmic vacuolization, blebbing, rounding up, and cellular detachment from the culture plates. This is similar to the morphology findings of Shrivastava and colleagues, who observed increased cellular vacuolization and nuclear condensation in MDA-MB-231 breast cancer cells treated with CBD [19]. Their findings demonstrated that CBD caused dose-dependent cell death, resulting in both apoptosis and autophagy. In this study, the morphology of cells at low concentrations did not differ from untreated cells, despite extended incubation periods. However, biochemical tests revealed that CBD exerts an effect even at the lowest concentrations over time. When cells are damaged and their membranes rupture, intracellular contents are released into the extracellular space, which for in vitro research is the culture medium. With increasing CBD concentrations beginning at 5 μg/mL and continuing for 24 h, LDH levels increased. Less than fifty percent of LDH was disseminated in the media. 12 h after incubation with 5 μg/mL CBD, the LDH results were not statistically significant; however, the ATP and trypan blue results indicated a significant decrease in cellular viability. Measuring LDH is a reliable indicator of the toxicity of compounds in cells. There is no established method for determining the precise time between the beginning of apoptosis and the rupture of the cell. In addition, the statistical error in LDH studies is substantial due to the nature of the enzyme and the methodologies employed. In data plotting and potency measurements, LDH is, therefore, more likely to provide an inaccurate EC_50_ value [20]. However, the assay is a useful indicator of general cytotoxicity if the purpose is checking response/effect rather than calculating statistical EC_50_/IC_50_ values.

It is essential to observe that LDH is an enzyme with a half-life ranging from 6 to 40 h, depending on the isoform. The half-life of the isoform in most assay media, including the CytoTox 96^®^ Non-Radioactive Cytotoxicity Assay used in this study, is 9 h [21,22]. This indicates that LDH can only be detected in the medium within nine hours of its discharge from cellular compartments. Nonetheless, it is necessary to know when cells begin releasing LDH into the media, a task made difficult by the fact that the duration of in vitro apoptosis from initiation to cell death is difficult to predict. Due to the absence of specific apoptotic proteins, signals, or channels, in vitro/ex vivo apoptotic cells will eventually endure secondary necrosis [23]. Depletion of ATP and a decline in cell population viability provided additional evidence of cellular injury. The lack of ATP in metabolically active cells indicates the absence of metabolism. The decreasing ATP concentration indicated the dose-dependent toxicity of CBD, which was corroborated by the trypan blue results. Trypan blue, like ATP, distinguishes viable cells from non-viable cells by exclusively staining non-viable cells. Because their cell membranes are intact and impermeable to the dye, living cells do not absorb the stain. Both the ATP and trypan blue results showed a statistically significant decrease in CBD-treated cells after a 12 h incubation, beginning with the lowest concentration and progressing to the highest, with 50% reduction at 5 μg/mL CBD treatment.

All these observations suggest that CBD has a chemotoxic influence on MCF-7 breast cancer cells. This effect of CBD is inferable in vivo, and is a viable alternative for inhibiting tumor cell proliferation. CBD is a naturally occurring compound that is abundant, easily accessible, and nontoxic to normal cells [24]. Numerous retail outlets around the world sell purified CBD oil, and numerous physicians already prescribe CBD oil for a variety of health conditions. Numerous studies have demonstrated the efficacy of CBD in the treatment of breast cancer [25,26,27] and other cancers, such as brain [28,29,30] and colorectal [31]. 

Comparable to this research, Schoeman and colleagues observed cytoplasmic vacuolization in their CBD-treated cells, which is one of the findings of this study. Schoeman and colleagues determined that the vacuole membranes were derived from the endoplasmic reticulum, which is the most active organelle during apoptotic cell death [32]. In addition, the novel use of CBD and hypericin as natural compounds for PDT increases the likelihood of enhanced cancer treatment options. According to the findings of this study, natural compounds can inhibit cancer cell proliferation and induce apoptosis. Studies showed that natural compounds have the capacity to inhibit CRC cell proliferation by inducing cell cycle arrest [33] or apoptosis [34], thereby inhibiting tumor growth. They observed that, in combination therapy, certain natural compounds can sensitize to conventional cytotoxic therapy, increase the drug’s effective concentration, amplify the combined effect of both administered therapeutics, and exert cytotoxic effects specifically on tumor cells. Similar results were observed in this study, in which the application of PDT followed by CBD increased cell mortality in MCF-7 cells by decreasing ATP levels. In addition, Rejhová and colleagues discovered that combined therapy which targets multiple signaling pathways reduces the emergence of antitumor drug resistance. This makes the use of natural compounds preferable to conventional therapies, which are associated with a number of unwanted adverse effects [35]. Many natural compounds are well tolerated by patients and do not cause toxic effects, even at high concentrations. The interaction of conventional chemotherapeutics with natural compounds introduces a novel aspect to the study of cancer therapy. It could be a promising strategy for achieving advances while minimizing the side effects of conventional cancer treatments. Apart from the breast cancer CBD and related compounds have shown potential in treatment of various other cancer types, in pilot clinical trials such as melanoma [36], leukemia [37], cervical [38], lung [39], prostate [40], colorectal cancers [41]. 

Immunofluorescence results from this study demonstrated that MCF-7 cells were dying via the apoptotic pathway. This was demonstrated by the lower expression of Bcl-2 relative to Bax and the higher expression of cytochrome c. Bcl-2 is renowned for its anti-apoptotic function during cell death, whereas Bax is categorized as a pro-apoptotic protein [38]. This is further corroborated by the expression of cytochrome c, which indicates that mitochondrial damage has led to the cytoplasmic release of cytochrome c, which induces apoptosis by activating caspases [42,43]. The Bax results are also consistent with the upregulated tumor suppressor protein p53 expression observed during apoptosis. Consequently, this indicates that the treatment induced apoptosis. The ability of p53 to induce apoptosis, cell cycle arrest, and senescence was observed in this research [44]. The presence of PARP-1 indicates that DNA repair is not occurring, and that therapy-induced cell mortality is irreversible. Shrivastava and co-workers also noted a significant increase in cleaved PARP and a decrease in Pro-PARP, which they concluded was a result of apoptosis induction. Similarly to their study, we also observed that CBD alone led to dose-dependent cell death, whereas the combination therapy led to apoptotic cell death, as confirmed by immunofluorescence results. 

Many studies have shown that cannabinoids can influence the rate of cell proliferation, migration, angiogenesis, and apoptosis, especially in breast, prostate, and glioma cell lines [45,46,47]. Studies have also revealed that the combination of CBD with PDT enhances the treatment efficacies in various cancers, such as metastatic melanoma [48], cervical cancer [49,50], colorectal cancer [51], etc. Similarly to these reports, this study showed the enhanced therapeutic potential of CBD and PDT in breast cancer cells by upregulating the activity of apoptotic proteins. Cannabinoids and cytotoxic medicines have been shown to have synergistic effects [52]. The addition of CBD to conventional chemotherapy with Paclitaxel merely boosted the antiproliferative effects in ovarian cancer cells and had no influence on the chemotherapeutic cytotoxic effect. Furthermore, CBD did not reduce Paclitaxel’s efficiency in inhibiting breast cancer cell viability [26,53]. 

## 5. Conclusions

In conclusion, PDT at very low concentrations sensitizes cells to CBD treatment for enhanced tumor destruction, which is advantageous for combination therapy at very low concentrations of drugs used. In addition, there is a high likelihood that the obtained results can be applied to the treatment of breast cancer. The results infer that the combined therapies have a passive mode of action that is effective against cancer cells due to their permeable vasculature and ability to absorb drugs effectively and/or sensitize the cells to respond to the therapies by synergistic effects. In order to gain a greater understanding of the Hypericin-AuNP drug delivery into deeply cantered tumors and the effect of CBD in such an environment, the results of this study ought to be investigated further using in vivo models and protein and gene expression profiling. This will have the intended effect of reducing the patient’s financial burden by substituting conventional chemotherapeutic interventions with natural substances that have well-defined effects. CBD and other cannabis derivatives, such as THC, still have a great deal of unexplored mechanisms, as the majority of their specific cellular and molecular mechanisms are not fully understood. Consequently, additional research on said mechanisms, alone and in combination therapy, is necessary. Despite control cells would be important to be added in this experiment, our main goal was to investigate the effect of CBD-AuNP-Hypericin Photosensitiser combination on hormone responsive MCF-7 Breast cancer cell line.

## Data Availability

Data is contained within the article.
